# Targeted mutagenesis of a thermophilic mevalonate kinase for efficient catalysis at lower temperatures

**DOI:** 10.1093/protein/gzag027

**Published:** 2026-09-29

**Authors:** Sylvia A Sarnik, Jeffrey N Law, Sam J B Mallinson, Hunter B Harrington, Joel L Kaar, Daniel K Schwartz, Yannick J Bomble

**Affiliations:** Department of Chemical and Biological Engineering, University of Colorado, Boulder, CO 80309, United States; Biosciences Center, National Laboratory of the Rockies, Golden, CO 80401, United States; Biosciences Center, National Laboratory of the Rockies, Golden, CO 80401, United States; Biosciences Center, National Laboratory of the Rockies, Golden, CO 80401, United States; Biosciences Center, National Laboratory of the Rockies, Golden, CO 80401, United States; Department of Chemical and Biological Engineering, University of Colorado, Boulder, CO 80309, United States; Department of Chemical and Biological Engineering, University of Colorado, Boulder, CO 80309, United States; Biosciences Center, National Laboratory of the Rockies, Golden, CO 80401, United States

**Keywords:** biocatalysis, thermophilic enzyme, mevalonate kinase, targeted mutagenesis

## Abstract

Thermophile-derived enzymes are of interest for cell-free biocatalysis due to their intrinsic thermostability, which simplifies purification *via* heat treatment and correlates with enhanced activity retention under cell-free reaction conditions. However, thermal stability often comes at the expense of slower turnover rates for reactions that need to be conducted at ambient temperature out of concern for cofactor stability and to reduce energy requirements. This study demonstrates a targeted engineering strategy to increase the room temperature activity of the thermophilic mevalonate kinase from *Methanocaldococcus jannaschii*. Site-saturation mutagenesis of evolutionarily variable residues within the mevalonate binding pocket generated numerous mutants with increased activity at 22°C. Fifteen mutants demonstrated a two- to four-fold increase in activity, and the most active mutant, I10S, achieved a five-fold increase in *k_cat_* relative to the wild-type. Importantly, the I10S mutant retained thermostability comparable to the wild-type, and a minimal decline in long-term stability was observed. This mutagenesis strategy may have broader applications towards tuning thermophilic enzyme activity at desired operating temperatures to enhance the efficiency of cell-free reactions while reducing operating costs.

## Graphical Abstract

**Graphical Abstract:**
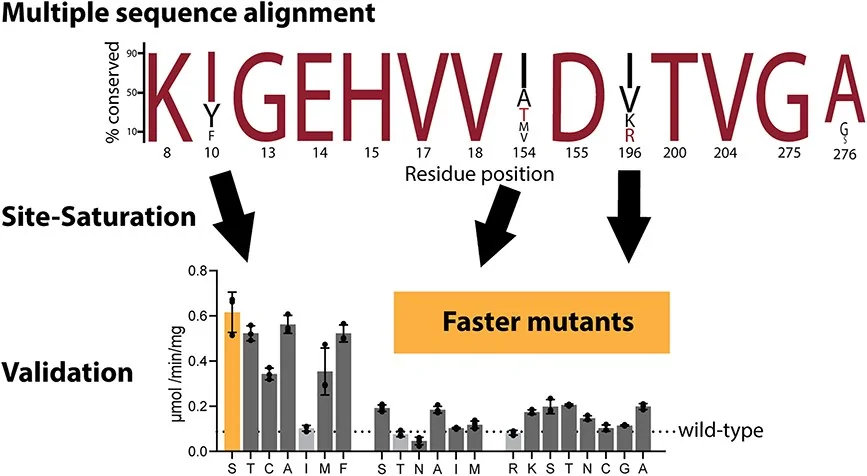

## Introduction

Isoprenoids are a diverse class of molecules with valuable applications ranging from pharmaceuticals, to alternative bioplastics and biofuels (George et al., 2015). There has been great interest in developing synthesis methods for isoprenoids to replace current production methods which involve either extraction from plants or using complex chemical syntheses (Quílez del Moral et al., 2020; Li et al., 2020). These existing methods often rely on toxic solvents and result in low yields due to the structural complexity of isoprenoids. In the last few decades, biocatalysis has developed as an alternative production strategy promising higher yields, cleaner processes, and more cost-effective synthesis methods for isoprenoid production.

Biocatalytic production of isoprenoids has traditionally emplo-yed the mevalonate pathway, derived from non-plant organisms, for production of isopentenyl pyrophosphate (IPP), a universal building block for isoprenoid products (Lange et al., 2000; Eisenreich et al., 2004). In bacteria and eukaryotes, the mevalonate pathway converts Acetyl-CoA, taken from glycolysis, to IPP (Lange et al., 2000). IPP is then isomerized to dimethylallyl pyrophosphate (DMAPP), and recombination of these building blocks results in highly varied isoprenoid, or terpene, products (Wang et al., 2019). Metabolic engineering has been applied towards the synthesis of diverse isoprenoids, with modest success, typically resulting in yields on the order of 0.01–1 g/L (Chang & Keasling, 2006; Immethun et al., 2013; Kang et al., 2024). Drawbacks of engineered whole-cell factories include competing pathways that limit yield, as well as the toxicity of intermediates or products, which results in limited production titers (Brennan et al., 2012; Chubukov et al., 2015; George et al., 2018). As an alternative to *in vivo* isoprenoid production, cell-free biocatalysis promises higher yields and mitigated toxicity concerns (GEN Biotechnology, n.d.). Foundational work by Rodriguez *et al.* demonstrated that cell-free reconstitution of the mevalonate pathway could produce IPP from mevalonate, at high concentrations up to 40 g/L (Rodriguez & Leyh, 2014). Further iteration since has resulted in titers of close to 15 g/L of monoterpene products starting from glucose, employing a pathway of over 25 enzymes, and demonstrating an order of magnitude improvement in titers as compared to *in vivo* systems (Korman et al., 2017).

Despite several cases of high titer production of isoprenoids through cell-free biocatalysis, industrial adoption of cell-free manufacturing is still limited. Enzyme instability poses a major challenge in cell-free catalysis, as denaturation results in loss of productivity and increased costs. Significant efforts have been dedicated to engineering mesophilic enzymes for improved stability (Shi et al., 2025; Liu et al., 2019). Engineering approaches, ranging from rational design to machine learning platforms such as PROSS, have been utilized to introduce stabilizing features such as hydrophobic core packing, hydrogen bonding, and backbone rigidity to improve enzyme stability (Goldenzweig et al., 2016). Stabilization efforts of mesophilic enzymes may be hindered by the stability-activity tradeoff, in which enhanced stability often results in reduced activity rates of the target enzyme (Liu et al., 2019). As an alternative starting point, enzymes derived from thermophilic organisms are of interest for cell-free systems, due to their evolved stabilizing features, such as denser core-packing and increased intermolecular bonding, which enable them to withstand extreme temperatures (Atalah et al., 2019; Littlechild, 2011). Thermotolerant enzymes enable rapid and scalable biocatalyst purification using heat treatment (Pessela et al., 2004; Ren et al., 2007). Beyond thermostability, the stabilizing features of thermophile-derived enzymes make them more resistant to other challenges found in cell-free catalysis (Atalah et al., 2019; Vieille & Zeikus, 2001). For example, thermophile-derived enzymes tend to be more resilient when challenged with solvents (Sherkhanov et al., 2020; https://www.sciencedirect.com/science/article/pii/S1369703x09002964  n.d.). Additionally, thermophilic enzymes may also retain activity for longer at moderate temperatures as compared to mesophilic counterparts, however experimental validation of this trend is still limited (Sarnik et al., 2025). Greater long-term activity retention would enable longer reaction lifetimes, recycling of the enzyme for multiple reactions, or even enabling continuous flow biocatalysis (Broumidis et al., 2025).

Despite offering numerous advantages, thermophile-derived enzyme turnover rates at ambient operating temperatures are typically lower compared to those of their mesophilic counterparts (Vieille & Zeikus, 2001; Bae & Phillips, 2004; Georlette et al., 2003). Often, ambient temperature is used for one-pot reactions to reduce energy demands, maintain cofactor stability, or ensure the stability of non-thermostable enzymes essential to the cascade (Sherkhanov et al., 2020). In such cases, the operating temperature may be significantly lower than the evolved temperature optima of the enzyme. This suboptimal performance necessitates higher enzyme loadings to match desired turnover rates, thereby increasing enzyme production burden and even potentially introducing colloidal instability (Mao et al., 2025).

Previously, the lower mevalonate pathway was ruggedized by replacing mesophilic enzymes with thermophilic variants, resulting in improved stability and productivity compared to the mesophilic pathway; however, the thermophilic enzymes exhibited slower reaction rates than the mesophilic enzymes (Sarnik et al., 2025). Specifically, the thermophile-derived mevalonate kinase from *Methanocaldococcus jannaschii* (MjMvk) was found to be five-fold slower at 22°C as compared to the mesophile-derived mevalonate kinase from *Methanosarcina Mazei* (*Sarnik et al., 2025*). To overcome this limitation, we investigated an enzyme engineering strategy for increasing the turnover rate of the thermophile-derived MjMvk at ambient operating temperatures.

Several efforts have focused on engineering thermophilic enzymes to increase their activity at ambient temperatures, using either directed mutagenesis or more targeted strategies (Li et al., 2017; Akanuma et al., 2019). For example, Akanuma et al. aligned the sequences of a thermophilic enzyme and its mesophilic counterpart, subsequently substituting active site residues in the thermophile with corresponding residues from the mesophilic enzyme. This approach required extensive mutagenesis within a broad radius around the active site to produce a more active mutant. Here, we employ an alternative strategy, focusing on a limited number of residue positions, and applying site-saturation mutagenesis to comprehensively explore the effect of all amino acid substitutions at each position. First, a multiple sequence alignment of archaeal mevalonate kinases was conducted, focusing on residues within a 5 Å radius of the mevalonate binding site, to identify binding-site residues that were not evolutionarily conserved. Site-saturation of these non-conserved residues resulted in identification of sixteen mutants with activity greater than the wild-type (WT) at 22°C. The most active mutant had over five-fold increased activity, ten-fold increase in *K_M_*, and only minimal destabilization as compared to the WT. The increased turnover rate of MjMvk at the desired operating conditions will serve to mitigate enzyme production burden and improve cell-free cascade efficiency.

## Results

### Identification of target mutagenic residues in the mevalonate binding pocket

The number of potential mutation targets was reduced by focusing on amino acids within the substrate binding pocket (approximately one hydration shell around mevalonate) that are not evolutionarily conserved, thereby minimizing the risk of altering essential catalytic residues. Site-saturation of each non-conserved residue position was then conducted to assess the range of functional diversity achievable.

Initially, a crystallized MjMvk structure (PDB: 1KKH) was aligned with a previously crystallized archaeal mevalonate kinase bound to mevalonate, from *Methanosarcina Mazei*, (PDB: 6MDE), to determine the likely mevalonate orientation within MjMvk (Fig. S1) (Yang et al., 2002; Miller and Kung, 2018). The alignment RMSD was 0.942, indicating high similarity between the structures. Residues located within 5 Å, approximately one hydration-shell, of the mevalonate molecule were identified as binding-site residues. Conservation of these 14 residues was then determined using multiple sequence alignment (MSA) of archaeal mevalonate kinases (Fig. 1). Over 2000 sequences from the UniProt Database were aligned utilizing the ClustalOmega program. Conservation of each residue was determined based on frequency of appearance at that position (Fig. 1B, Fig. S2) (Madeira et al., 2024a). The WT MjMvk sequence has a lysine at residue position 8. In the MSA, lysine was found to be very highly conserved, as it appeared in approximately 90% of sequences at this equivalent position. In contrast, position 10 exhibited more amino acid diversity. Isoleucine was the most frequently observed residue at this position; however, tyrosine and phenylalanine also appeared in a substantial portion of sequences. A cutoff of <70% evolutionary conservation for non-conserved residues was selected to allow mutagenesis of residues with evolutionary bias (>50% conservation) while minimizing the risk of altering essential catalytic residues, which are likely to be highly conserved. Residues at positions 10, 154, and 196 exhibited less than 70% conservation, classifying them as non-conserved. Consequently, these residues were selected as targets for mutagenesis, shown in blue in Fig. 1C. Having identified the non-conserved residues in the mevalonate binding pocket, site-saturation mutagenesis was performed on these targets to evaluate their impact on the catalytic activity of MjMvk.

**Figure 1:**
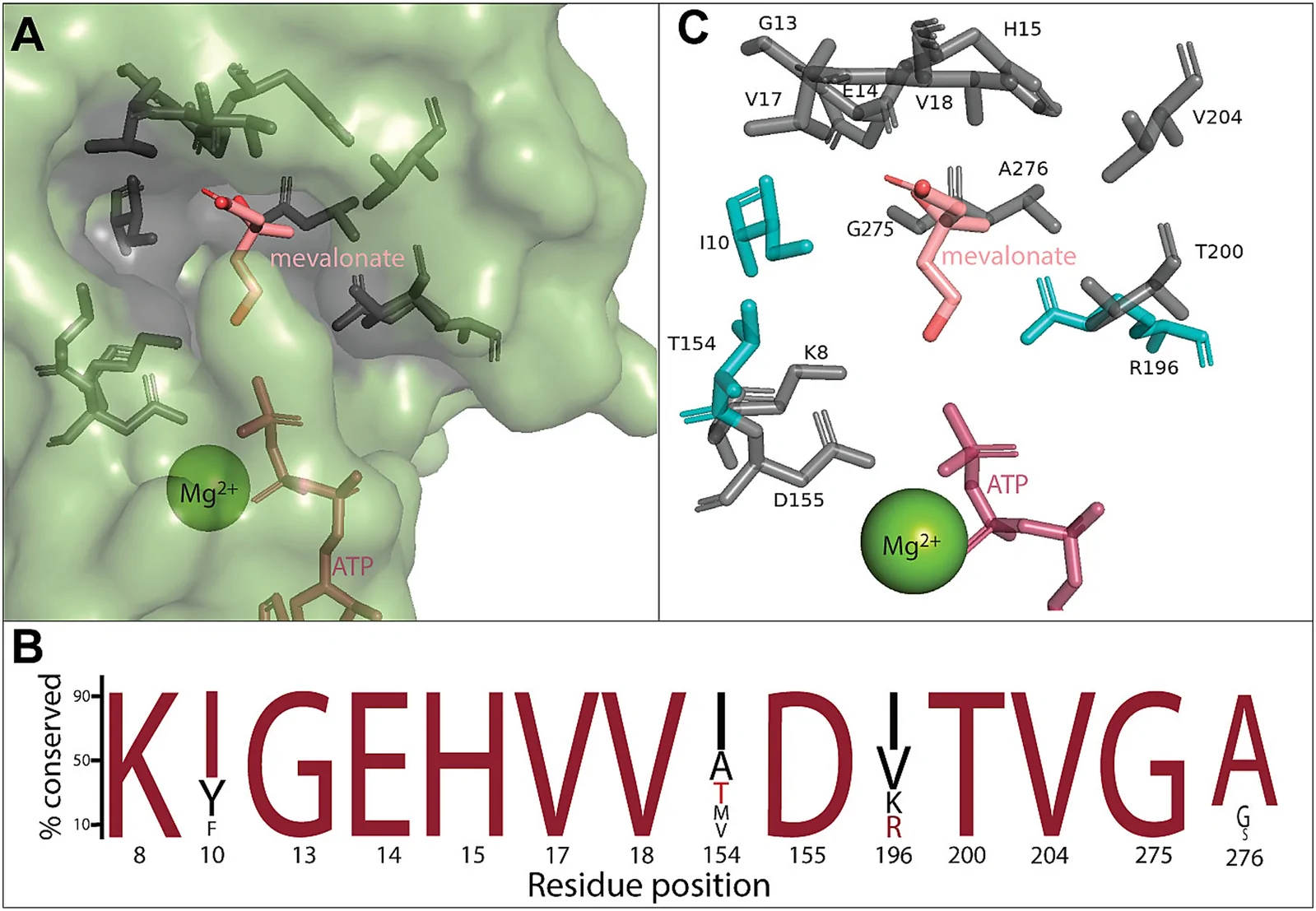
Identification of non-conserved residues in the MjMvk substrate binding pocket. **A.** Binding pocket of MjMvk, residues within the first hydration shell (5 Å) are depicted. **B.** Conservation of residues within the mevalonate binding pocket (approximate depiction, see Fig. S2 for exact percentages). Residues in the MjMvk WT sequence at each position are indicated in red. **C.** Mevalonate binding pocket residues, with non-conserved residues of interest shown in blue.

### Screening and validation of MjMvk site-saturation mutants with improved activity at ambient temperature

Site-saturation mutagenesis, and subsequent high-throughput screening, was performed on the non-conserved residues of MjMvk at positions 10, 154, and 196. Heat treatment of the expression cell lysate eliminated background activity from native *E. coli* enzymes (data not shown) and mevalonate kinase activity was assayed by ATP utilization assay for phosphorylation activity of mevalonate. Notably, for each site-saturated position, there were several substitutions with activity greater than the WT (Fig. 2A-C). Substitution of I10 to S, T, C, A, M, or F resulted in up to a three-fold increase in activity. Substitutions for T154 to S, N, A, I, or M resulted in mutants with activity greater than the WT, with S, N, or I demonstrating roughly three-fold activity increase over the WT. R196 substitutions to K, S, T, N, C, G, or A resulted in a modest increase in activity, with the cysteine substitution approaching a two-fold increase in activity over WT. Because the variants were screened in lysate, the initial activity measurements did not account for potential differences in enzyme concentration. As such, all mutants with activity greater than WT were considered potential hits and selected for further validation.

**Figure 2:**
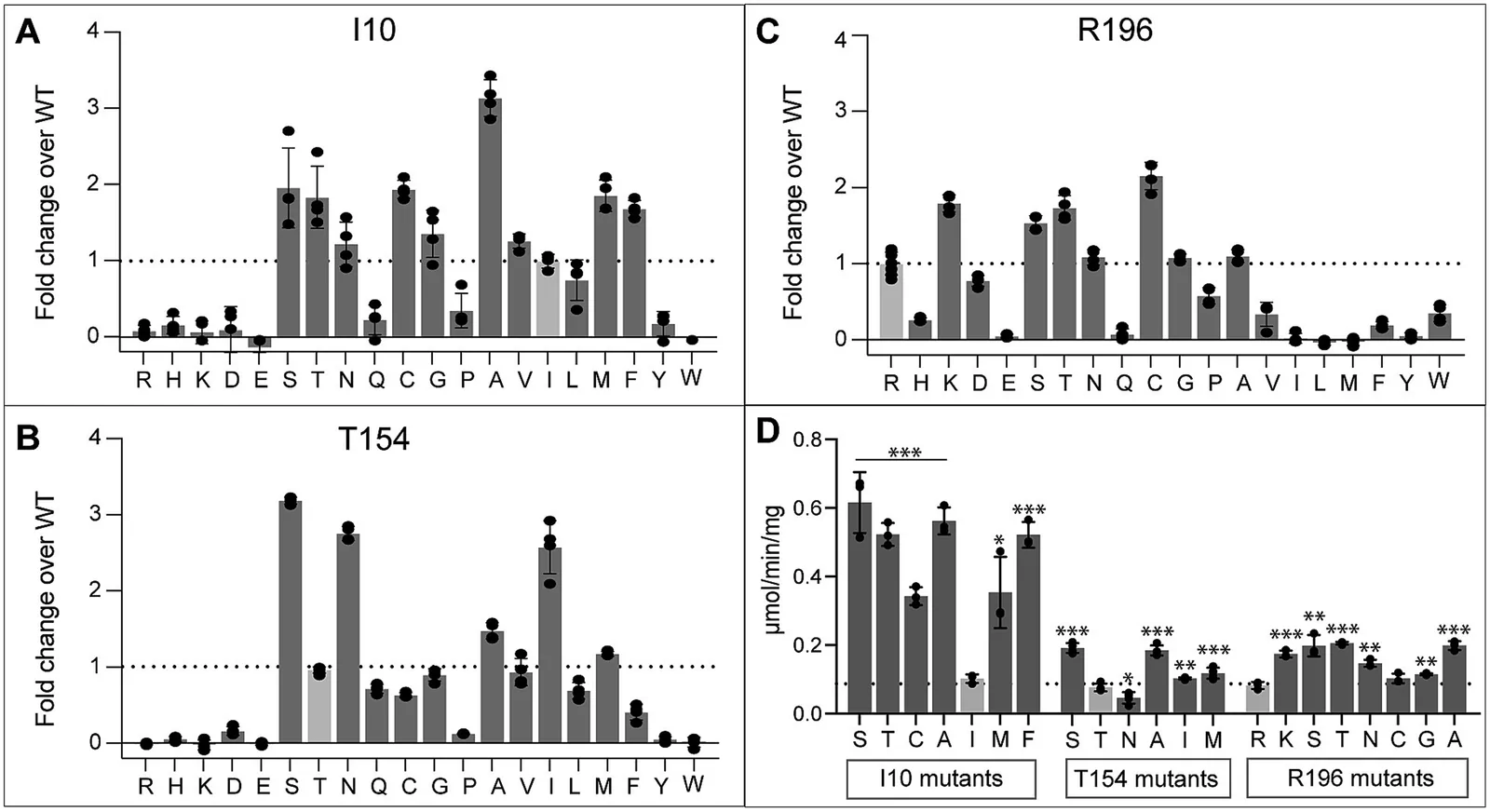
Screening of site-saturation mutants at positions 10, 154, and 196. **A-C**. Fold change activity over the WT when heat-treated lysate was assayed for mevalonate kinase activity. Dashed line as well as lighter color bar indicate the WT activity from each testing batch. **D.** Specific activity of MjMvk mutants assayed at 22°C. Dark grey bars indicate activity rates of mutants; light grey bars indicate activity of the WT. dashed line indicates the average activity of all WT samples tested. T-tests were used to compare each mutant to the WT within that batch, * = p < 0.05, **p = <0.01, *** = p < 0.001.

The variants were subsequently validated by comparing the specific activity of the purified mutants at 22°C (Fig. 2D**)**. Most of the potential hits identified in the high-throughput screening demonstrated improved specific activity rates compared to the WT; only one variant, T154N, exhibited a lower activity than the WT. Residue substitutions at position 10 had the greatest impact on activity rates; substitutions to serine, threonine, alanine and phenylalanine resulted in an over four-fold increase in activity. Mutations at positions 154 and 196 had a more modest effect, with top mutants resulting in approximately two-fold increases in activity. Serine and alanine were the top substitutions for residues T154 and R196, respectively. Altogether, through identification of non-conserved residues, and subsequent site-saturation, a mutant with a five-fold increase in activity over WT was identified.

Having validated the abovementioned mutations, we subsequently explored if combinations of these mutations had additive or synergistic effects to further improve the activity of MjMvk. Various attempts at combining mutations at positions 10, 154 and 196 of MjMvk did not prove fruitful in producing a more active mutant (Fig. 3). Combinations of the top mutations (I10S, T154S, R196A) as pairwise mutants, or all in combination, generally resulted in decreased activity as compared to the single I10S mutant (Fig. 3A). Interestingly, most of the double mutations, as well as the triple mutation, were deleterious, with the triple mutant exhibiting almost no kinase activity. An alternative approach attempted to evolve the I10S mutant through site-saturation at position 154. Double mutants with I10S and T154 substituted to K, S, A, and M had similar activity to the I10S single mutant, however this approach did not generate any mutants with significantly greater activity than the I10S mutant itself (Fig. 3C).

**Figure 3:**
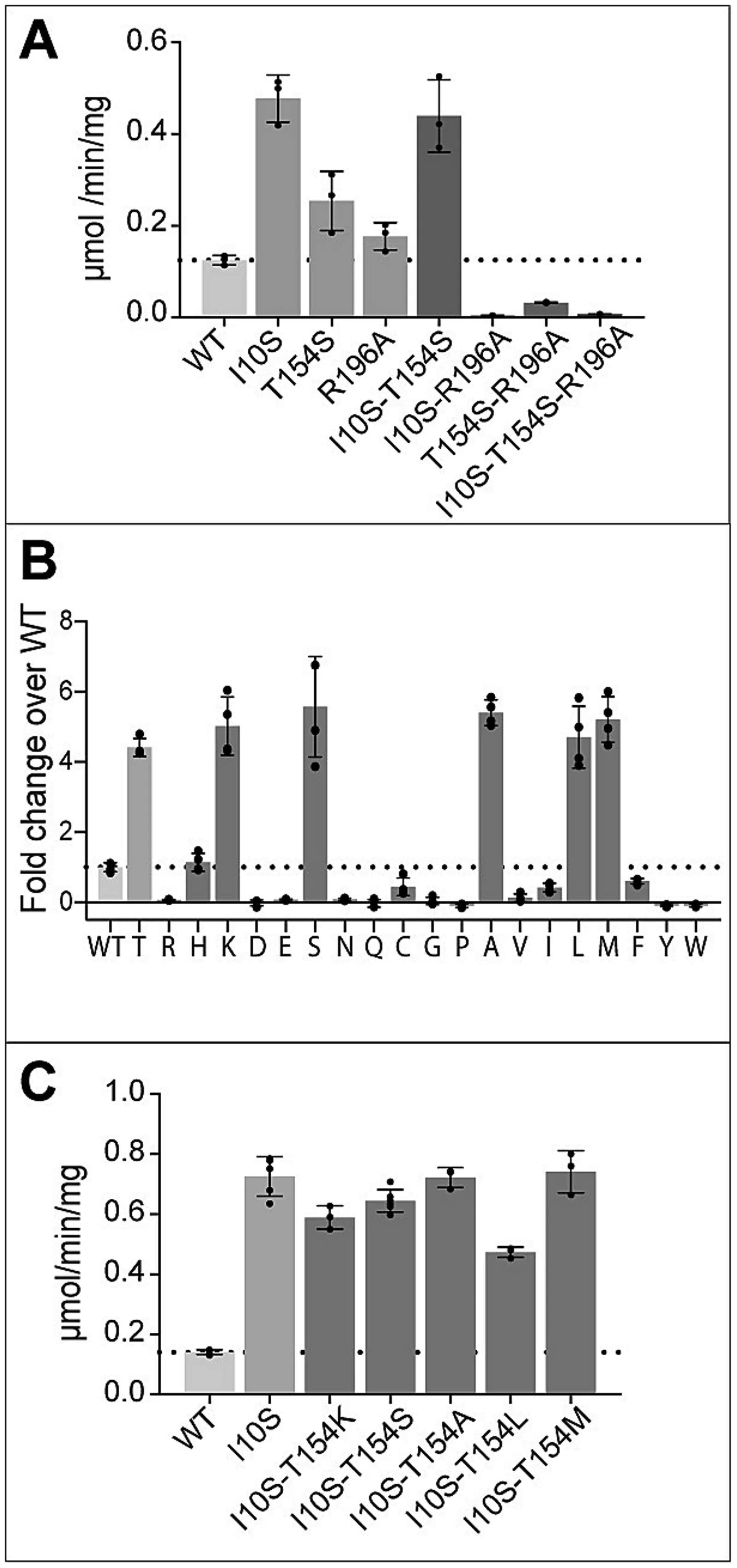
Combination of mutations **A.** combining top mutations at each position. **B.** Site saturation of position 154, using the I10S mutant as the parent. **C.** Validation of the mutants from site-saturation of position 154 on the I10S mutant.

### MjMvk-I10S retains WT-like thermostability and exhibits substantial retention of long-term activity

Thermostability and long-term stability were compared between the WT and I10S mutant MjMvk. Figure 4A shows activity remaining after a one-hour incubation at temperatures up to 80°C. Upon heating to 65°C there was an approximate 30% activity loss in both the WT and mutant. Activity rates remained at ~70% after incubation at 70 and 75°C. At 80°C both the mutant and WT activities decreased to below 50%. Importantly, the I10S mutation did not impact thermostability; I10S MjMvk retained similar relative activity as WT MjMvk after incubation at various temperatures.

**Figure 4:**
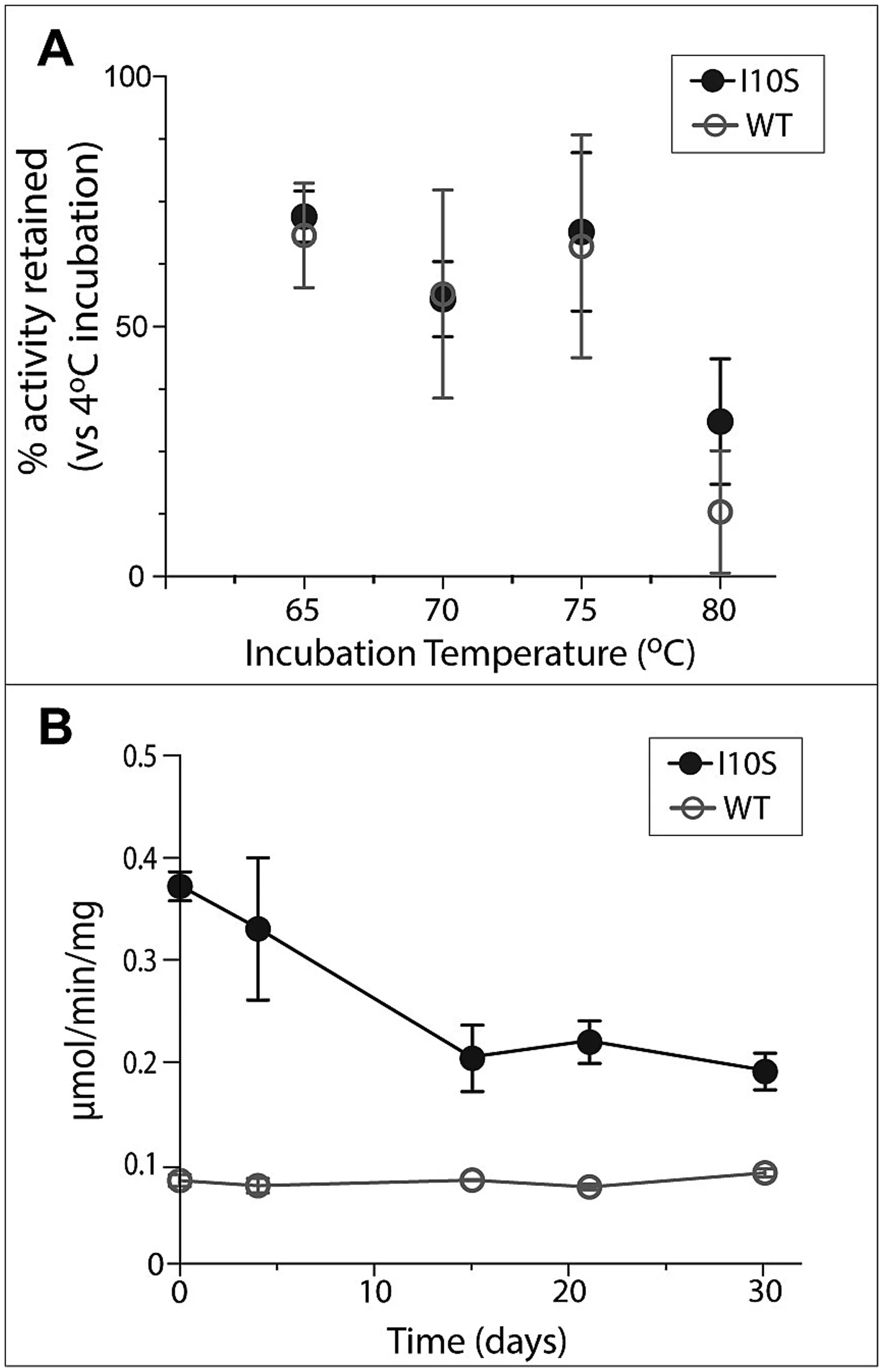
Stability of the I10S mutant *vs* WT MjMvk. **A.** Activity retention after 1-hour incubation at elevated temperatures, as compared to a 4°C incubated control. T-test between mutant and WT at each temperature found no significant activity difference. **B.** Specific activity of the WT and I10S mutant after incubation at 22°C for up to 30 days.

Long-term stability (*i.e.* activity retention) of an enzyme is also of great interest, as it indicates lifetime productivity potential, as well as possibility for enzyme recycling. To characterize long-term (*i.e.* kinetic) stability, the I10S mutant and WT MjMvk were incubated at room temperature for 30 days and periodically assayed for activity over time (Fig. 4B). Notably, the I10S mutant exhibited reduced long-term relative activity retention, losing approximately 50% of its activity after two weeks of incubation, whereas the WT retained 100% activity. Despite this decline in activity over time, the I10S mutant consistently maintained a specific activity that was over two-fold higher than the WT throughout the 30-day testing period.

### Characterization of I10 mutants

The I10 mutants, substituted with serine or alanine, were further characterized. Expression analysis in *E. coli* demonstrated that both variants were produced at levels comparable to the WT enzyme, with His-tag purification yielding approximately 50 mg/L (Fig. S3). Michaelis–Menten parameters of these enzymes were measured at 22°C to determine the effect of mutating residue 10 (Fig. S4). Table 1 indicates the *K_M,_* and *k_cat_* values for tested mutants. The *k_cat_* for the I10S mutant was 0.485 s^−1^, approximately five-fold faster than the WT with a *k_cat_* of 0.096 s^−1^. The I10A mutant had an intermediate *k_cat_* of 0.361 s^−1^. Interestingly, *K_M_* values followed a similar trend. The I10S mutant had the highest *K_M_*, 0.21 mM mevalonate, I10A had a *K_M_* of 0.12 mM, and lastly the WT had the lowest *K_M_* of 0.02 mM mevalonate. Altogether, the fastest mutant, MjMvk-I10S, demonstrated a 5-fold increase in *k_cat_* compared to the WT, achieving turnover rates comparable to a mesophile-derived mevalonate kinase from *Methanosarcina Mazei* which had been previously employed in several cell-free reactions (Korman et al., 2017; Sarnik et al., 2025)*.* The corresponding increase in *K_M_*, from 0.02 mM to 0.2 mM for the I10S mutant, is not a concern for cell-free reactions where substrate abundance is not expected to be limiting. Moreover, a *K_M_* value below 1 mM remains well within the *K_M_* range typically observed in enzymes (ranging from 0.0001–100 mM) (Berg et al., 2002). To contextualize the performance of this new variant, a summary of previously reported mevalonate kinases is provided in Table S3.

**Table 1:** Michaelis–Menten parameters for I10 mutants and WT MjMvk assayed at 22°C. ± values indicate SEM for specific activity and *K_M_*.

	**I10S**	**I10A**	**WT**
Specific activity (μmol/min/mg)	0.83 ± 0.053	0.62 ± 0.026	0.16 ± 0.01
*k_cat_* (s^−1^)	0.485	0.361	0.096
*K_M_* (mM)	0.21 ± 0.052	0.12 ± 0.024	0.02 ± 0.015
*k_cat_* (s^−1^) /*K_M_* (mM)	2.3	3.0	4.8

## Discussion

### Developing a targeted engineering strategy for increasing MjMvk activity at 22°C

Increasing the turnover rates of the thermophile-derived MjMvk under ambient operating conditions could improve cell-free biocatalytic efficiency by decreasing enzyme production burden. To this end, we employed a site-saturation mutagenesis approach targeting non-conserved residues within the substrate-binding pocket, aiming to identify variants with enhanced catalytic activity. Residues within the mevalonate binding pocket were identified based on proximity to mevalonate, specifically within a 5 Å (or one-hydration shell) radius. Expanding the radius of residues around mevalonate, could help reveal more beneficial mutations, however at the cost of increasing experimental burden. With the 5 Å radius applied in this study, 14 residues were identified as ‘binding site residues’ and potential targets for mutagenesis.

Conservation analysis of the binding-site residues with an MSA of MjMvk against other archaeal mevalonate kinases revealed 93% conservation of the aspartic acid at position 155 in MjMvk. D155 is a well-established catalytic residue essential for coordinating the magnesium ion during mevalonate phosphorylation activity (Yang et al., 2002; McClory et al., 2019). This finding (*i.e.* D155 exhibiting <100% conservation) was expected, as some sequences in the Uniprot database are truncated, and there is a possibility of incorrect classifications as mevalonate kinases (Table S1). Nonetheless, the strong conservation of this key residue confirmed the accuracy of the high-throughput alignments and conservation analysis. Performing an MSA across thousands of mevalonate kinases (all archaeal sequences available in UniProt) allowed for the identification of residues with subtle plasticity, such as I10 with 58% conservation, which may not be apparent when aligning fewer sequences, as is standard for MSAs.

To select non-conserved residues, the <70% conservation cutoff allowed for mutagenesis of evolutionarily biased residues (>50% conserved). However, the upper limit of 70% minimized chances of mutating catalytically necessary residues. Future work investigating higher limits for conservation (*e.g.* mutating residues less than 80%, or 90% conserved) could help determine the upper limit where conservation directly relates to catalytic necessity. Using the <70% cutoff for conservation, residues I10, T154 and R196 were found to be non-conserved, and thus the final targets for mutagenesis. It was unexpected that R196 was a non-conserved residue, as a previously proposed mechanism by Yang *et al.* suggested this residue was involved in hydrogen-bonding with mevalonate (Yang et al., 2002; Chu et al., 2003). The other non-conserved residues, I10 and T154 had not been previously implicated as having a role in MjMvk activity.

### Mutation of non-conserved active site residues generates many faster MjMvk mutants

Site-saturation of non-conserved residues in the MjMvk substrate binding pocket resulted in identification of at least three mutants at each position with increased activity over the WT (Fig. 2). These results indicate an advantage of this approach. Investigating single amino acid substitutions at each position, as proposed by Akanuma et al. to replace residues with those from a mesophilic counterpart enzyme, would have greatly limited the number of more active mutants identified (Akanuma et al., 2019). Most notably, I10 mutations had the biggest impact on mevalonate kinase reaction rates. The low conservation score of 58% indicates the isoleucine itself is not crucial to catalysis (Fig. S2). However, the markedly increased turnover rates observed in several different mutants at this position suggest that it may play an important role in the active site, such as in substrate binding or orientation, which warrants further study.

Interestingly, combining mutations at positions 10, 154 and 196 resulted in mostly deleterious double mutants and an inactive triple mutant (Fig. 3A). It is possible that combining these top mutations results in incorrect binding orientation or otherwise prevents binding of the substrate rendering the enzyme inactive. Furthermore, when the I10S mutant was used as the parent for site-saturation mutagenesis of the T154 position, no mutants were identified with significantly improved activity over the I10S mutant (Fig. 3B-C). It was not entirely surprising that mutations within the binding pocket may not have cooperative effects that result in increased activity rates. Epistatic effects of mutations have been previously described, especially when mutating several residues within a small distance of each other (Starr & Thornton, 2016; Khersonsky et al., 2018). Given the elusiveness of cooperative mutations, our approach may be limited to the identification of beneficial single mutants; however, this has the advantage of reducing the likelihood of destabilizing the protein by minimizing the number of mutated residues. For engineering targets requiring greater activity improvements, machine-learning approaches such as FuncLib, designed for active site optimization, could help identify cooperative combinations of mutations without imposing an immense *in vitro* experimental burden (Khersonsky et al., 2018). Alternatively, high-throughput screening methods or selections may be necessary to enable massive engineering campaigns.

### I10S mutation increased turnover rates with minimal impact on stability

Characterization of stability of the I10S mutant confirmed that thermostability of the enzyme was not affected. No significant difference in response to heat treatment indicates the I10S mutation did not disrupt the thermostable structures in MjMvk (Fig. 4A) (Li et al., 2005). This retention of stability is critical for enabling the use of high-throughput, heat-purification methodologies. Long-term activity retention at ambient incubation temperatures was evaluated as a proxy for operating lifetime of the biocatalyst. This metric highlights the potential of MjMvk, or the I10S variant, for extended production runs, reuse across multiple batch reactions, or even enabling flow reactions without the need for constant replenishment of fresh enzyme. The long-term incubations at 22°C revealed the I10S mutant had lower activity retention over time compared to the WT. Importantly, despite the decline in activity over time, the I10S mutant retained over two-fold higher specific activity as compared to the WT, for up to 30 days of incubation. As such, the overall productivity of the I10S mutant would still surpass that of the WT for at least 30 days of use.

Kinetic analysis of the I10S mutant revealed a five-fold increase in *k_cat_* and a ten-fold increase in *K_M_* relative to the wild-type enzyme. Comparing these parameters to other mevalonate kinases is challenging due to the limited availability of kinetic data at 22°C (Table S3). Currently, the only reported value is for the mesophilic *M. mazei* mevalonate kinase, which exhibits a *k_cat_* of 1 s^−1^ at 22°C, approximately twofold higher than that of the I10S mutant (Sarnik et al., 2025).

In contrast, other mesophilic mevalonate kinases typically achieve *k_cat_* values between 10 to 30 s^−1^ (at temperatures of 25 to 37°C) and *K_M_* values ranging from 0.02 to 0.12 mM. These values correspond to catalytic efficiencies *k_cat_ / K_M_* of 60 to 1080 s^−1^ mM^−1^. By comparison, the I10S mutant displayed a more modest *k_cat_ / K_M_* of 2.3 s^−1^ mM^−1^when measured at 22°C. As the characterization of thermophile-derived mevalonate kinases remains limited, the I10S variant exhibits, to the best of our knowledge, the highest turnover rate for a thermophilic mevalonate kinase at this temperature. Given that thermophile-derived enzymes are essential for workflows involving heat-purification, this variant represents a valuable catalyst for the development of cost-effective cell-free production platforms.

**Figure 5:**
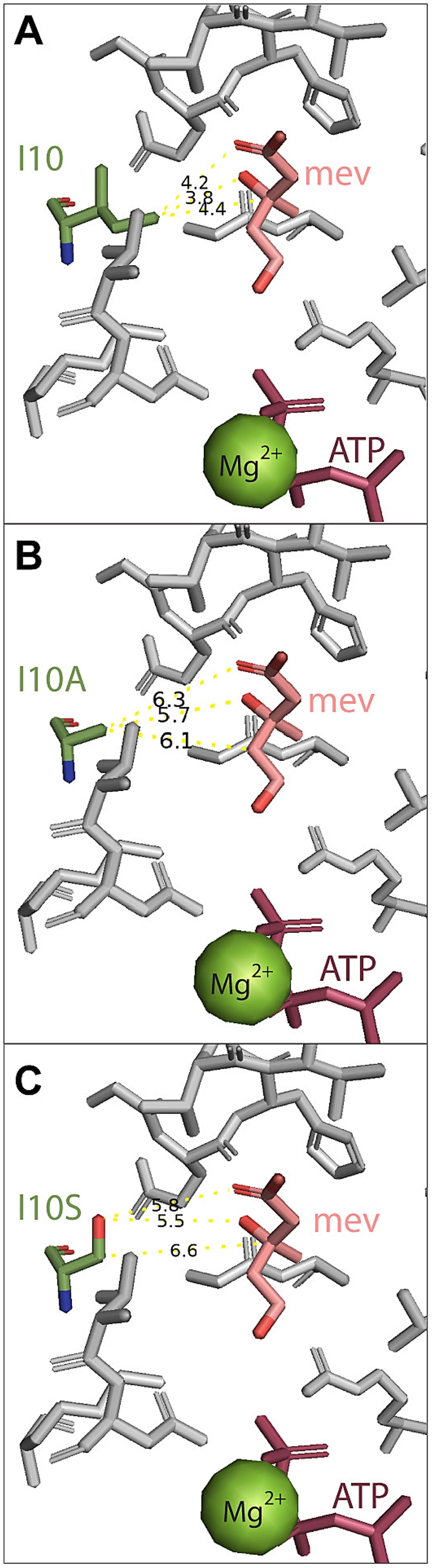
MjMvk mevalonate binding site in the WT, I10A and I10S mutants. Mevalonate shown in pink, Mg^2+^ in green, ATP in maroon, and other residues in the 5 Å radius in grey. (PDB: 1KKH aligned to PDB: 6MDE).

The mechanism underlying the increased activity rates in I10 mutants was investigated by measuring enzyme kinetics and *in silico* analysis. In many cases, increased activity at low temperatures can be attributed to increased enzyme flexibility, at the cost of thermal stability (Isaksen et al. 2016). However, the I10S mutant displays higher catalytic efficiency without sacrifice of thermal stability. Thus, the targeted strategy of mutating non-conserved residues preserved global stability of the enzyme, suggesting the apparent increased activity is due to an alternate mechanism. One possibility is that mutations at the I10 residue modify the enzyme-substrate binding dynamics, facilitating more efficient substrate-product exchange at 22°C. This may be explained by the evolution of WT MjMvk to bind tightly to mevalonate to counteract entropic barriers to substrate binding at its optimal operating temperature of 70°C. A similar trend has been observed in enzymes derived from psychrophiles (organisms that grow at temperatures below 20°C), which tend to exhibit higher *K_M_* values compared to enzymes from mesophiles (Gerday, 2013). Importantly, high affinity for the substrate may relate to increased affinity for the product, which in turn reduces substrate-product turnover. This mechanism is consistent with the apparent increase in *K_M_* for the I10S mutant compared to WT. It is possible that the decreased substrate affinity of the I10S mutant may enable more efficient turnover at ambient temperatures, where entropic forces are reduced. Alternatively, the I10S mutation may affect activity by altering solvation of the active site or changing the substrate binding orientation. To elucidate if the mechanism of the apparent activation of the I10 mutants at ambient temperature was due to changes in the binding of mevalonate, structures for I10A and I10S were generated *in silico* and compared with WT MjMvk (Fig. 5). Minimal distances between the position 10 residue and mevalonate are indicated for each mutant. Isoleucine has the closest interaction to mevalonate, at 3.8 Å. It is possible that some hydrophobic interactions facilitate proper orientation of the substrate in the active site. The I10A mutant also presents a hydrophobic moiety, however the residue is smaller, now interacting with mevalonate at a distance of 5.7 Å. This lessened hydrophobic interaction may decrease affinity for mevalonate, thus resulting in the intermediate phenotype of I10A. Lastly, the I10S mutant has a minimum distance of 5.5 Å between polar groups on the serine and mevalonate molecule. At this distance it is possible repulsive forces are destabilizing the binding of mevalonate, resulting in the increased *K_M_* and corresponding higher *k_cat_*.

## Conclusions

In conclusion, a targeted engineering strategy focusing on non-conserved residues within the substrate binding pocket increased ambient-temperature activity in a thermophile-derived enzyme. This approach generated 16 mutants with activity greater than WT MjMvk. However, a limitation of targeting a small region, such as the substrate binding pocket, is the potential for epistatic interactions between mutations. While numerous highly active single mutants were identified, combining top mutations did not yield cooperative effects. The most active single-mutant, I10S, demonstrated a five-fold increase in *k_cat_* at 22°C without compromising thermostability. This improvement in catalytic rate, at the desired operating temperature, reduces the enzyme production demand by lowering the enzyme quantity needed per cell-free reaction. While we have demonstrated the utility of this approach for MjMvk, further work is necessary to understand how broadly applicable this engineering approach is for other thermostable enzymes. Ultimately, this approach may have significant implications in improving the overall efficiency of cell-free biocatalytic processes that leverage the enhanced stability of thermostable enzyme variants and moreover, may have application in enhancing the utility of thermostable enzymes in other applications, including environmental biosensing.

## Methods

### Identification of non-conserved residues of interest

The crystallized MjMvk structure (PDB: 1KKH) was aligned with an archaeal mevalonate kinase bound to mevalonate (PDB: 6MDE) to identify the probable binding site and orientation of mevalonate within MjMvk (Fig. S1). A 5 Å radius around mevalonate in MjMvk was used to identify residues of interest for mutagenesis. The list of residues was K8, I10, G13, E14, H15, V17, V18, T154, D155, R196, T200, V204, G275, A276. Next, a multiple sequence alignment (MSA) was performed to identify conservation of these residues.

For the MSA, Archaeal mevalonate kinase sequences were downloaded from UniProt, consisting of 2867 entries. Alignment of all these sequences was performed using the ClustalOmega online program (https://www.ebi.ac.uk/jdispatcher/msa/clustalo) (Madeira et al., 2024b). The alignments were reformatted from FASTA to a table, and duplicate sequences were removed, resulting in 2269 aligned sequences remaining (Table S1). A custom MATLAB script was then employed to calculate percent conservation at each residue of interest.


\begin{align*} &\%\ \mathrm{conservation}\nonumber\\&\quad=\frac{\mathrm{number}\kern0.17em \mathrm{sequences}\kern0.17em \mathrm{containing}\kern0.17em \mathrm{the}\kern0.17em \mathrm{residue}\kern0.17em \mathrm{of}\kern0.17em \mathrm{interest}}{\;\mathrm{total}\kern0.17em \mathrm{number}\kern0.17em \mathrm{of}\kern0.17em \mathrm{sequences}} \end{align*}

### 2‌2C-trick mutagenesis of MjMvk

Methodology of Kille *et al.* was followed (Kille et al., 2013). Briefly, PCR was performed using primers shown in Table S2. A 50 uL PCR reaction containing 5 ng of MjMvk DNA, 10 uM of 3 mixed forward primers, 10uM reverse primer, 25 uL of 2× Q5 master mix (NEB), and nuclease-free water (Invitrogen) to final volume was ran according to Q5 PCR cycles, with Tm at 65°C. Post-PCR DNA was digested with DPN1 (Thermo Fisher Scientific) for one hour at room temperature. The PCR-product was run on a 1% agarose gel, stained with SYBR safe DNA gel stain (Thermo Fisher Scientific), and the expected band size of ~6000 bp was excised then gel-extracted with the GeneJET kit (Thermo Scientific). DNA was eluted in 4 uL of nuclease-free water, then mixed with 4 uL of 2× KLD reaction buffer and 0.8 uL of KLD enzyme mixture (NEB). After a 20-minute incubation at room temperature, the DNA mixture was heat-shock transformed into 50 uL of BL21 (DE3) cells (Thermo Fisher scientific). After a one-hour recovery incubation at 37°C the cells were plated on Kanamycin (50 ug/mL, Kan50) LB agar plates for selection. The next day individual colonies were plated in a 96-well for high throughput protein expression or for downstream sequencing by LevSeq.

### LevSeq

Post 22C-Trick mutagenesis, surviving colonies after selection were picked and cultured in a 96-well plate overnight. 2 μL of overnight starter culture was used as a template for the LevSeq PCR (Long et al., 2025). Methodology of Long *et al.* was followed for the PCR and subsequent sample processing. The pooled post-PCR sample from all 96 wells was sequenced by Plasmidsaurus. The Long *et al.* LevSeq program was used to demultiplex the barcodes and assign mutations to each well.

### Enzyme expression

The gene encoding for MjMvk (UniProt: Q58487) was cloned into a pET28 backbone, conferring Kanamycin resistance. The plasmid was transformed into *E. coli* BL21(DE3) cells (ThermoFisher) for expression. Starter cultures were seeded from plated colonies into Kan50 media overnight at 37°C and rpm 220. The following day, expression culture was scaled up with 1% volume of the starter and incubated at 37°C, rpm 220. OD600 was monitored until reaching 0.6–0.8. Protein expression was induced with addition of IPTG to a final concentration of 0.2 mM, and flasks were returned to the incubator at 18°C overnight. After 16–20 hours the culture was centrifuged, media was discarded and the pellet was frozen at −20 until future lysis for protein purification.

For 96-well expressions- 5% of overnight starter culture was used to inoculate scaleup culture. After 3 hours at 37°C, 900 rpm, protein expression was induced with IPTG at a final concentration of 0.2 mM. Plates were then returned to the incubator at 18°C. After 16–20 hours, the plates were centrifuged, media was discarded, and pellets were frozen at −20 until future lysis.

### Enzyme purification

The frozen cell pellet was suspended in HisA buffer (25 mM imidazole (Millipore-Sigma), 400 mM NaCl (FisherScientific), and 50 mM HEPES (Millipore-Sigma) at pH 7.5) and sonicated on ice. The sample was centrifuged at 4500 g for 10 min. The supernatant was then heated in an oven at 65°C for 90 minutes while mixing by inversion. The heat-treated sample was then centrifuged at 15000 g ×15 minutes. Supernatant was then loaded onto an Akta Pure Chromatography (AKTA) system for His-trap purification with a 5 mL HisTrap FF (Ni-NTA resin, Cytiva) column. Elution was performed with HisB buffer (300 mM imidazole, 150 mM NaCl, and 50 mM HEPES at pH 7.5). Eluted protein was directly loaded onto a Sephadex G-25 desalting column (Cytiva) to desalt with GF buffer (50 mM HEPES at pH 7.5 and 100 mM NaCl). Eluted fractions were pooled, concentrated with Amicon 10KDa MWCO columns (Millipore-Sigma), and supplemented with glycerol (Millipore-Sigma) to a final concentration of 10%. Enzyme concentration was assayed by Bradford Plus Protein Assay Kit (Pierce), and then samples were aliquoted and flash frozen on liquid nitrogen before storage at −20°C.

### High throughput activity testing

Enzyme pellets from 96-well expressions were thawed in 300 μL/well GF buffer supplemented with 2 μL/50 mL universal nuclease (Pierce), and 0.5 mg/ mL chicken-egg lysozyme (ThermoFisher). Suspended pellets were incubated at 37°C for 30 minutes and shaking at 225 rpm, then heat-treated at 65°C for 1.5 hours and shaking at 900 rpm. Post heat-treatment, the 96-well plate was centrifuged at 4000 rpm ×30 minutes at 4°C. The clarified lysate was transferred to a fresh plate and diluted 10-fold with GF buffer. 50 μL of the diluted clarified lysate was added to mevalonate kinase assay buffer as described above, and absorbance at 340 nm was tracked for 1 hour with 10 second timepoints.

### Long-term stability

Enzymes were diluted in GF buffer to 1 mg/mL. Triplicate vials of each enzyme (WT and I10S) were incubated in the dark at 22°C. At indicated timepoints, a sample was taken and frozen at −80°C, then stored frozen until all timepoints were complete. Upon completion of the time-course, all samples were thawed and activity was tested in one plate to minimize assay variability.

### Activity assay

A coupled assay using pyruvate kinase and lactate dehydrogenase (Pyk-Ldh mixture, Sigma-Aldrich) was used to track ATP consumption based on NADH (reduced nicotinamide adenine dinucleotide) oxidation resulting in a decrease of absorbance at 340 nm. Assay concentrations per 150 μL reaction included 0.5 mM ATP (Millipore-Sigma), 3 mM phosphoenolpyruvate (Roche), 0.7 mM NADH (Fisher Scientific), 6 mM base-treated mevalonolactone (effectively 2 mM bioavailable mevalonate (Sarnik et al., 2025)), 0.75 μL of Pyk-Ldh, 30 mM HEPES pH 7.5, 15 mM MgCl_2_ (Millipore-Sigma), 5 mM KCl (Millipore-Sigma), and 0.5 mM MnCl (Millipore-Sigma). All assays were performed in triplicate (n = 3) using a SpectraMax190 plate reader.

For Michaelis–Menten (MM) kinetic analysis the I10S and I10A mutants were assayed at 0.01 mg/mL concentration. To approximate similar rates, 0.028 mg/mL of the WT MjMvk enzyme was used for MM experiments. In each well of a 96-well plate, a 120 μL mixture was prepared with the Mvk enzyme, 0.5 mM ATP, 3 mM phosphoenolpyruvate, 0.7 mM NADH, 0.75 μL of Pyk-Ldh, 30 mM HEPES pH 7.5, 15 mM MgCl_2_, 5 mM KCl, and 0.5 mM MnCl. To this mixture, 30 μL of dilutions of base-treated mevalolactone were added to a final concentration of 0–8.58 mM in the 150 μL final volume (effectively a range of 0–2.6 mM mevalonate). The spectrophotometer was immediately started to capture initial kinetic rates. Mevalonate concentration was plotted against initial specific activity rate (Fig. S4) and GraphPad Prism’s non-linear regression fit was used to calculate MM parameters for *K_M_*, and *V_max_* from which *k_cat_* could be calculated.

## Supplementary Material

Supporting_information_gzag027

TableS1_gzag027

## Data Availability

The data underlying this article will be shared upon reasonable request to the corresponding author.
