# Supplementary material for: Targeted mutagenesis of a thermophilic mevalonate kinase for efficient catalysis at lower temperatures
Source: Protein Eng Des Sel. 2026 Sep 29;39:gzag027. doi: 10.1093/protein/gzag027 (PMC13654298; doi:10.1093/protein/gzag027)
Supplement: Supplementary file 1 [file supporting_information_gzag027.docx]

**Supporting information**

Table S1: Aligned archaeal mevalonate kinase sequences (Attached Excel spreadsheet).

Table S2: Primers for site-saturation mutagenesis employing 22C-Trick.

Table S3: Comparison of mevalonate kinase kinetic parameters.

Figure S1: Alignment of MjMvk and MmMvk crystal structures

Figure S2: Conservation of active-site residues

Figure S3: Post purification yields of MjMvk variants

Figure S4: Michaelis Menten graph for I10 mutants

| **Table S2:** PCR primers for 22C-Trick mutagenesis | | |
| --- | --- | --- |
| **Target site** | **Mutation Codon** | **Primer** |
| I10 | NDT | NDTTTATTCGGGGAGCACG |
|  | VHG | VHGTTATTCGGGGAGCACG |
|  | TGG | TGGTTATTCGGGGAGCACG |
|  | Reverse | AACTTTGGATGGGGTTTC |
| T154 | NDT | AGCTAGTATCNDTGACACGAG |
|  | VHG | AGCTAGTATCVHGGACACGAG |
|  | TGG | AGCTAGTATCTGGGACACGAG |
|  | Reverse | TTCCCTTGGATCTCCTTTTC |
| R196 | NDT | GCAGAGAAGNDTAAGAAGAAG |
|  | VHG | GCAGAGAAGVHGAAGAAGAAG |
|  | TGG | GCAGAGAAGTGGAAGAAGAAG |
|  | Reverse | GTAAACGATTAAGAATTTGC |

| **Table S3**: Comparison of mevalonate kinase kinetic parameters. Assay temperature is indicated in parenthesis for each measurement, N.R. indicates a measurement was not reported. | | | |
| --- | --- | --- | --- |
| Source Organism  (Thermophile shown in red) | K_cat_ (s^-1^) | K_M_ (mM) | K_cat_/K_M_ |
| *M. jannaschii* | 0.096 (22^o^C)^1^  190 (70^o^C)^2^  29 (34^o^C)^3^ | 0.02 (22^o^C)^1^  0.068 (70^o^C)^2^  0.01 (34^o^C)^3^ | 4.8 (22^o^C)^1^  2794 (70^o^C)^2^  2900 (34^o^C)^3^ |
| *M. jannaschii*  MjMvk-I10S | 0.49 (22^o^C)^1^ | 0.21 (22^o^C)^1^ | 2.3 (22^o^C)^1^ |
| *M. Mazei* | 1 (22^o^C)^4^  4.3 (30^o^C)^5^  10 (25^o^C) ^6^ | N.R.  0.07 (30^o^C) ^5^  0.12 (25^o^C) ^6^ | N.R.  61 (30^o^C) ^5^  81 (25^o^C) ^6^ |
| *M. burtonii* | 5 (25^o^C)^6^ | 0.055 (25^o^C)^6^ | 90 (25^o^C)^6^ |
| *S. aureus* | 6.6 (30^o^C)^7^ | 0.041 (30^o^C) ^7^ | 160 (30^o^C) ^7^ |
| *H. Sapiens* | 25.9 (30^o^C) ^8^ | 0.024 (30^o^C) ^8^ | 1080 (30^o^C) ^8^ |
| *R. norvegicus* | 27.09 (37^o^C)^9^ | 0.035 (37^o^C)^9^ | 774 (37^o^C)^9^ |
| 1. This work 2. Huang KX, Scott AI, Bennett GN. Overexpression, purification, and characterization of the thermostable mevalonate kinase from Methanococcus jannaschii. *Protein Expr Purif*. 1999;17(1):33-40. doi:10.1006/prep.1999.1106 3. Chu X, Yu W, Wu L, Liu X, Li N, Li D. Effect of a disulfide bond on mevalonate kinase. *Biochim Biophys Acta BBA - Proteins Proteomics*. 2007;1774(12):1571-1581. doi:10.1016/j.bbapap.2007.09.004 4. Sarnik SA, Martinsen MR, Korman TP, Schwartz DK, Kaar JL, Bomble YJ. Thermostable Enzyme Variants in the Lower Mevalonate Pathway Improve Isoprenoid Production by Cell-Free Biocatalysis. *ACS Sustain Chem Eng*. 2025;13(32):12971-12980. doi:10.1021/acssuschemeng.5c03763 5. Primak YA, Du M, Miller MC, et al. Characterization of a feedback-resistant mevalonate kinase from the archaeon Methanosarcina mazei. *Appl Environ Microbiol*. 2011;77(21):7772-7778. doi:10.1128/AEM.05761-11 6. Esquirol L, Newman J, Nebl T, et al. Characterization of novel mevalonate kinases from the tardigrade Ramazzottius varieornatus and the psychrophilic archaeon Methanococcoides burtonii. *Acta Crystallogr Sect Struct Biol*. 2024;80(3):203-215. doi:10.1107/S2059798324001360 7. Voynova NE, Rios SE, Miziorko HM. Staphylococcus aureus Mevalonate Kinase: Isolation and Characterization of an Enzyme of the Isoprenoid Biosynthetic Pathway. *J Bacteriol*. 2004;186(1):61-67. doi:10.1128/jb.186.1.61-67.2004 8. Potter D, Miziorko HM. Identification of Catalytic Residues in Human Mevalonate Kinase*. *J Biol Chem*. 1997;272(41):25449-25454. doi:10.1074/jbc.272.41.25449 9. Chu X, Li D. Expression, purification, and characterization of His20 mutants of rat mevalonate kinase. *Protein Expr Purif*. 2003;32(1):75-82. doi:10.1016/S1046-5928(03)00221-3 | | | |

**Figure S1:** Structural alignment of MjMvk (PDB: 1KKH) and a mevalonate kinase from *Methanosarcina Mazei* (MmMvk) which had been crystalized with mevalonate bound (PDB: 6MDE). MmMvk shown in purple, MjMvk in green and the mevalonate molecule in red.

**Figure S2:** Alignments of MjMvk active-site residues. Y-axis indicates the residue position in MjMvk. X-axis lists all amino acid residues. Darker squares indicate a higher percentage of sequences contain a certain residue at the indicated position. Bolded squares indicate the residue in WT MjMvk at that position.

**Figure S3:** Post His-tag purification yields of MjMvk variants from *E. coli* flask cultures.

**Figure S4:** Michaelis-Menten analysis of MjMvk WT and I10 mutants at 22 ^o^C. Mevalonate kinase activity rates were assayed at various concentrations of mevalonate.
